# Dynamics of the complex food environment underlying dietary intake in low-income groups: a systems map of associations extracted from a systematic umbrella literature review

**DOI:** 10.1186/s12966-021-01164-1

**Published:** 2021-07-13

**Authors:** Alexia D. M. Sawyer, Frank van Lenthe, Carlijn B. M. Kamphuis, Laura Terragni, Gun Roos, Maartje P. Poelman, Mary Nicolaou, Wilma Waterlander, Sanne K. Djojosoeparto, Marie Scheidmeir, Agnieszka Neumann-Podczaska, Karien Stronks, on behalf of the PEN Consortium

**Affiliations:** 1 Department of Public and Occupational Health, Amsterdam University Medical Centres, University of Amsterdam, Room J2-211, Meibergdreef 15, Amsterdam, 1105 AZ The Netherlands; 2 Department of Public Health, Erasmus Medical Centre, Rotterdam, 3000 CA The Netherlands; 3 Department of Interdisciplinary Social Science, Utrecht University, Utrecht, 3584 CH The Netherlands; 4 Department of Nursing and Health Promotion, Oslo Metropolitan University, Oslo, Norway; 5 Consumer Research Institute, Oslo Metropolitan University, 0170 Oslo, Norway; 6 Department of Social Sciences, Wageningen University, Wageningen, 6706 KN The Netherlands; 7 Department of Human Geography and Spatial Planning, Faculty of Geosciences, Utrecht University, Utrecht, 3584 CB The Netherlands; 8 Psychology Institute, Johannes Gutenberg University Mainz, D-55122 Mainz, Germany; 9 Department of Geriatrics and Gerontology, Poznan University of Medical Sciences, Poznań, Poland

**Keywords:** Diet, Low-income groups, Inequality, Food environment, Complex adaptive systems, System dynamics

## Abstract

**Background:**

Inequalities in obesity pertain in part to differences in dietary intake in different socioeconomic groups. Examining the economic, social, physical and political food environment of low-income groups as a complex adaptive system – i.e. a system of multiple, interconnected factors exerting non-linear influence on an outcome, can enhance the development and assessment of effective policies and interventions by honouring the complexity of lived reality. We aimed to develop and apply novel causal loop diagramming methods in order to construct an evidence-based map of the underlying system of environmental factors that drives dietary intake in low-income groups.

**Methods:**

A systematic umbrella review was conducted on literature examining determinants of dietary intake and food environments in low-income youths and adults in high/upper-middle income countries. Information on the determinants and associations between determinants was extracted from reviews of quantitative and qualitative studies. Determinants were organised using the Determinants of Nutrition and Eating (DONE) framework. Associations were synthesised into causal loop diagrams that were subsequently used to interpret the dynamics underlying the food environment and dietary intake. The map was reviewed by an expert panel and systems-based analysis identified the system paradigm, structure, feedback loops and goals.

**Results:**

Findings from forty-three reviews and expert consensus were synthesised in an evidence-based map of the complex adaptive system underlying the food environment influencing dietary intake in low-income groups. The system was interpreted as operating within a supply-and-demand, economic paradigm. Five sub-systems (‘geographical accessibility’, ‘household finances’, ‘household resources’, ‘individual influences’, ‘social and cultural influences’) were presented as causal loop diagrams comprising 60 variables, conveying goals which undermine healthy dietary intake.

**Conclusions:**

Our findings reveal how poor dietary intake in low-income groups can be presented as an emergent property of a complex adaptive system that sustains a food environment that increases the accessibility, availability, affordability and acceptability of unhealthy foods. In order to reshape system dynamics driving unhealthy food environments, simultaneous, diverse and innovative strategies are needed to facilitate longer-term management of household finances and socially-oriented practices around healthy food production, supply and intake. Ultimately, such strategies must be supported by a system paradigm which prioritises health.

**Supplementary Information:**

The online version contains supplementary material available at 10.1186/s12966-021-01164-1.

## Background

Non-communicable diseases (NCDs) such as cardiovascular disease, cancer and type 2 diabetes are estimated to account for 70% of all deaths worldwide, half of which are premature [1]. Obesity is a risk factor for NCDs and in Europe, individuals of a lower socioeconomic status carry the highest burden of obesity-related NCDs [2]. Poor dietary outcomes in low-income groups are likely to contribute substantially to a social gradient in the rates of overweight and obesity and associated health outcomes [3–5].

Evidence indicates that low-income groups are differentially exposed and vulnerable to the conditions that are associated with poorer dietary outcomes [6]. Although recent reviews of the literature report that individual-level factors such as food knowledge, beliefs and habits predict variation in dietary outcomes [7], there is also strong associative evidence for the role of the food environment [7]. The food environment encompasses social, physical, economic and political factors and can be characterised along four dimensions: food availability, affordability, accessibility and acceptability on a local or (inter)national scale [7–9]. Inequalities in dietary outcomes are suggested to stem partly from differential exposure and increased vulnerability to adverse food environments. For example, evidence shows poorer relative access to healthy foods (i.e. fresh, unprocessed and nutrient-rich foods) in low-income neighbourhoods and increased vulnerability to the cost of healthy foods for households on a low income [10–14]. As not all households on a low-income live in low-income neighbourhoods (and not all households in a low-income neighbourhood are on a low income), there are potentially important distinctions between determinants influencing dietary intake directly through income or indirectly through living in a low-income neighbourhood.

Despite the observed relationship between adverse food environments and poor dietary intake, it has been difficult to unpack the underlying mechanisms of this relationship in low-income groups, hindering the implementation of environmental interventions and policies that are acceptable and effective. For example, a modelling study using data from the United States reported that when accessibility and affordability of healthy foods was equal for lower- and higher-income households, the difference in dietary quality was reduced by only 10% [15]. The model suggested that the vast proportion of the difference was driven by demand, as opposed to food supply [15]. Such findings might explain why the introduction of new supermarkets in deprived neighbourhoods have reported a negligible effect on dietary outcomes [16]. However, qualitative research into the strategies used by low-income households to obtain sufficient or high quality dietary intake suggests that the situational, social, cultural and economic underpinnings of ‘demand’ are highly complex [17–19].

There is growing consensus that we can conceptualise the phenomenon of dietary intake in low-income groups as an emergent property of a complex adaptive system which has the following components and characteristics [20, 21]:

- *Elements* (determinants) are connected; change in one *element* or *connection* will affect other parts of the system;
- *Feedback loops* operating between system elements result in non-linear connections between elements: feedback can lead to growth or decline (i.e. *reinforcing feedback*) or can have a stabilising effect (i.e. *balancing feedback*);
- *Structure* formed by sub-systems with interconnected elements and feedback loops;
- Overarching *paradigm* which is represented by the *goals* which the system works to achieve; the system can adapt over time and self-organise in order to overcome minor modifications to the system and continue to work towards original system *goals* that are aligned with the *paradigm*.

Understanding the dynamics (i.e. connections and structure) of the system of environmental and individual determinants of poor dietary intake in low-income groups gives insight into how poor dietary intake may be sustained or reinforced. This understanding can inform strategies that prioritise the modification of system dynamics so that a healthier food environment might be the emergent outcome of the system, rather than an unhealthy food environment [22].

Within the field of systems dynamics, causal loop diagramming is a specific method that has proven useful to understand complexity, including in relation to obesity and diet [23]. It is not yet clear whether the wealth of literature studying environmental influences on food intake can be directly translated into a causal loop diagram. Therefore, the objective of this study was to develop and apply causal loop diagramming methods to systematically synthesise existing evidence in order to identify the system dynamics that sustain and reinforce a food environment that influences dietary intake in low-income groups. We proposed that the system would be arranged around four dimensions of the food environment: accessibility, availability, affordability and acceptability [8] and consequentially sought evidence of the factors shaping the food environment at the micro- (individual and social factors), meso- (neighbourhood factors) and macro-level (economic and political factors).

## Method

### Study design

A systematic umbrella review was conducted to provide the body of evidence from which the systems map was constructed. The systems map was presented as a series of causal loop diagrams (CLDs) [24]. As stated, a CLD is a tool used to present the dynamics of a complex adaptive system; as such, it is concerned with mapping the reinforcing and balancing feedback loops which, respectively, reinforce or sustain certain behaviours [24]. The review protocol was registered on Open Science Framework (https://osf.io/fm4xv).

### Systematic umbrella review

The umbrella review adopted an explanatory approach, which is better suited to questions about why a phenomenon exists than other approaches to literature reviews which are better suited to making a judgement on hypothesised, pre-specified relationships [25]. Systematic differences in the investigation of distal and proximal dietary determinants (e.g. public transport and cooking skills, respectively) were anticipated, both in their conceptualised integration in a wider system and the type of evidence used for identification. Specifically, when examining distal or latent constructs with indirect influences on diet, evidence is more likely based on observational or qualitative research; evidence supporting the role of proximal or observable constructs is more readily drawn from experimental or quantitative research. Therefore, the application of traditional hierarchies of evidence may divert attention towards particular dynamics of the system, depending on the inherent properties of the implicated determinants rather than their association with the outcome.

We based our approach on: conventional approaches to systematic database searching, existing recommendations for realist reviews [25] and recent discussion around developing systems-based reviews [26]. The novel *synthesis* of these approaches, for this study, allowed the formulation of an explanatory research question around deepening an understanding of relevant system dynamics, adoption of a pluralist approach to study selection by study design and evidence type and the creation of original data extraction and presentation processes which allowed us to elucidate system properties [25, 26]. Petticrew et al. [26] stress the importance of defining the scope of the research question. The scope can be considered the boundaries of the system under study and therefore pertain to the characteristics of the studied population, outcomes and determinants.

#### Population

Low-income populations in high- and upper-middle income countries (HUMIC), according to World Bank classifications, were included. Populations in lower-middle and low-income countries (LLMIC) were excluded, as associations between socioeconomic factors and dietary outcomes are not always consistent with associations in HUMIC due to broader macro level factors (e.g. economy, welfare provision). If reviews included data from across HUMIC and LLMIC, only data pertaining to HUMIC were included; if it was not possible to disaggregate findings, the review was excluded. A wide geographical scope was intended to capture a rich evidence base and ensure sufficient variation across contexts to support the detection of associations. The intended population was low-income groups, encompassing low-income households and households living in low-income neighbourhoods; where this information was not available, a proxy variable (e.g. educational level) was deemed appropriate where a low income or financial strain was explicitly noted as a characteristic of the population.

#### Outcomes

Literature was searched for the following outcomes and determinants; when variables were included in the systems map the distinction between outcomes and determinants was not made as they were all treated as interconnected variables.

Outcomes at an individual level included those pertaining to food choice, eating behaviour and dietary intake, as specified in the taxonomy of outcomes in the *Determinants of Nutrition and Eating* (*DONE*) framework [27]. Foods not supporting a healthy diet are foods and drinks that are energy-dense, nutrient-poor and/or ultra-processed, comprising high levels of added sugar, saturated fats and/or salt, hereafter referred to as ‘unhealthy foods’. Food choice pertains to decisions and actions preceding the consumption of food (e.g. purchasing decisions and behaviours); eating behaviour pertains to the act of consumption (e.g. frequency of consumption, portion size); dietary intake pertains to the act of food consumption (e.g. (un)healthy food intake, meal pattern and food components) [27]. In line with the *DONE* outcomes taxonomy, food choice and eating behaviour were conceived as preceding dietary intake [27]. Environmental level factors that were treated as outcomes in the source reviews include those pertaining to: availability (food supply in the community and consumer environment), accessibility (location and consequent ease of access to the food supply), affordability (cost and perceived value of food) and acceptability (attitudes towards food supply and accommodation of the food supply to consumers’ requirements) [8].

#### Determinants

Socio-ecological dietary determinants were organised according to the *DONE* framework [27], encompassing individual (biological, demographic, psychological and situational), social, cultural, physical environmental and economic factors. If food choice and dietary behaviour factors were treated in the literature as determinants of dietary intake, they were also considered, including: preparation, purchasing and disordered eating [27]. Although policy factors are part of the *DONE* framework, they were not considered in the review as they were seen as potential interventions in the system, of which effects would be highly context-dependent, and therefore difficult to incorporate into the more generic system developed in this study. Food production and commercial determinants were also not within the scope of this review for reasons of feasibility and resources. Nonetheless, because these are important determinants of the food environment and likely influence food environments in low-income groups, in our results we acknowledged their importance by conceptualising their place within the wider system as part of a system overview.

#### Search strategy and record selection

A systematic database search was designed to obtain relevant systematic, non-systematic, scoping and mapping reviews of quantitative or qualitative research. The database search was performed in April/May 2019 (with the initial search on 10th April 2019) using key words in OVID (Medline, Embase) and Web of Science. There were no restrictions on the date of publication.

**Table:** 

Diet*, healthy eating, feeding, fruit*, vegetable*, fat* AND Determinant*, correlate*, concept*, construct*, underlying, map* AND Low-income, low income, disadvantage*, depriv*, socioeconomic, socio-economic, SES, income AND Review* or umbrella	

#### A-priori inclusion criteria

- Low-income (or proxy) sample, analysis by income (or proxy), or disaggregated findings reported for low-income (or proxy) groups;
- Sample of adults (> 18 years), children (> 2 years; upper bound determined by screened literature) and/or adolescents (≤18 years; lower bound determined by screened literature);
- Review of quantitative or qualitative observational or intervention studies/natural experiments examining determinants of dietary intake (including intake of specific foods, e.g. fruit and vegetables) and/or the food environment;
- (Non-)systematic, scoping, mapping review or meta-analysis, explicitly presented as a review of peer-reviewed scientific literature;
- High−/upper-middle-income countries;
- Determinants, correlates, predictors or intervening variables in the relationship between income (or proxy) and dietary intake;
- Determinants discussed in terms of income (or proxy); reporting differences in dietary intake by income (or proxy) is insufficient.

#### A-priori exclusion criteria

- Reviews of studies examining intervention techniques;
- Population groups with specific health-related issues (e.g. serious chronic illnesses; excluding pregnancy);
- Breastfeeding or other infant-specific dietary outcomes;
- Malnutrition by underweight; disordered eating symptoms; neophobia, pickiness, fussiness;
- Grey literature.

**Fig. 1:**
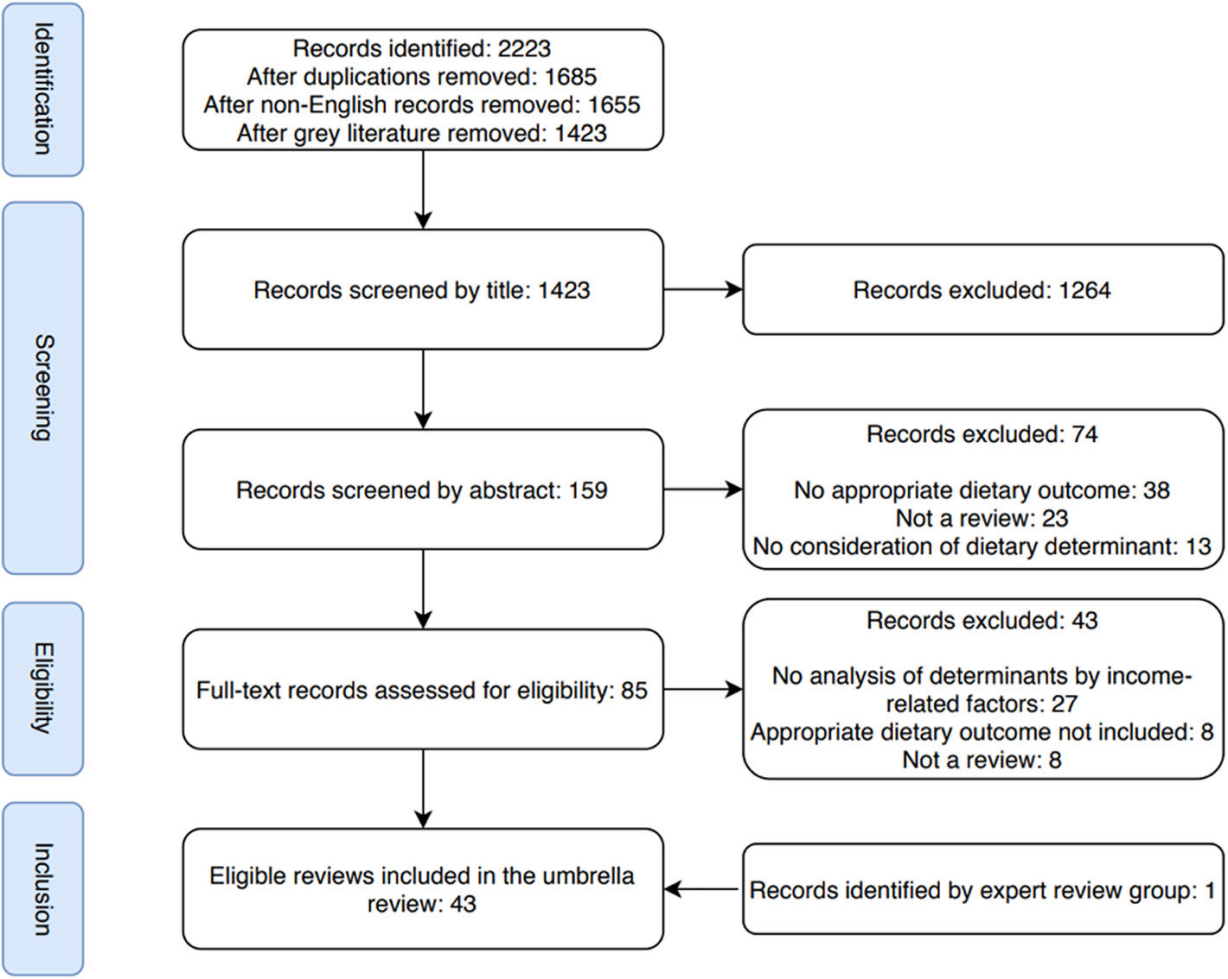
Flow chart of record screening and selection

Screening of record title, abstract and full-text against inclusion and exclusion criteria was performed by AS. All records were double-screened by KS at title and abstract stages and a random selection of 10% of records were double-screened by CBMK at full-text stage; any disagreements were resolved through discussion. Figure 1 presents the flow of record selection.

#### Quality appraisal

As recommended for exploratory reviews investigating mechanisms (e.g. system dynamics) [25], the current review was pluralistic in its inclusion of different study designs and review types (systematic, non-systematic, scoping and mapping). Therefore, the following methods for assessing risk of bias and confidence in the evidence were developed and reviewed by ADMS, KS and FvL, based on existing checklists for quality appraisals of systematic reviews (*ROBIS* and *AMSTAR.2*) and the *RAMESES* guidelines for assessing quality in realist reviews [28].

Specifically, the quality of each selected review was assessed in terms of ‘relevance’ and ‘rigour’ in addressing the topic and making methodologically credible inferences [25]. Quality assessments pertained to sections of each review which were relevant to our research question, rather than for each review in its entirety (for example, where a focus on low-income groups was only afforded limited attention). Selected reviews were screened according to the criteria below; only those which met the criteria were eligible for inclusion in the review. Quality appraisal found that all selected reviews met these criteria and were eligible for inclusion.

##### Relevance

- Alignment with inclusion/exclusion criteria;
- Consideration of appropriateness or representativeness of sample or population.

##### Rigour

- Appropriate justification for including/excluding individual studies within the review, e.g. in order to test a specific theory or research question (study selection; publication bias);
- Consideration of evidence quality from individual studies (quality appraisal; assessment of risk of bias);
- Consideration of the above-mentioned elements when interpreting the results of the review;
- Appropriate analytic methods to synthesise literature.

#### Data extraction

Sections of the included reviews that were relevant to the research question were highlighted and determinants, outcomes and any modifying variables were extracted. ADMS and CBMK categorised determinants and modifying variables using the *DONE* framework, first by *DONE* category (e.g. ‘social influence’) and then, if further specification was possible, by *DONE* variable (e.g. ‘social relationships’) [27]. If there was no appropriate *DONE* variable, a new variable was created (e.g. ‘stigma’). If further specification was not possible, then the *DONE* category was used as the variable label.

Next, these variables were transferred to a data matrix with *DONE* determinants down the first column and *DONE* outcomes and environmental outcomes along the top row. Information was extracted on the presence and direction of associations. In a quantitative review, all relevant associations were extracted with information on the direction and significance of each association according to the significance level used in the original study or review; this encompassed associations between determinants and outcomes as well as associations between determinants tested in analyses with effect modification (e.g. association between population density and walkability in an analysis examining a modifying effect of walkability in relationship between population density and dietary intake). Effect sizes were not recorded. In qualitative reviews, information on the stated, assumed pathways of causation was extracted (e.g. living in an area with low walkability increased spending on public transport to reach food outlets which reduced the available budget for shopping). Due to the volume of evidence and limited resources, it was not possible to go back to primary studies to extract data. Information on the source review for each data point was retained to enable linkage.

For each populated cell of the matrix, it was possible to ascertain the overall presence and direction of an association, presence of modifying variables and volume of supporting evidence. Where there was conflicting evidence in terms of presence or direction of an association, the overall relationship was determined by whichever was most commonly reported: significant or non-significant association at the significance level used by authors or as interpreted in qualitative literature. There were no instances where the balance of evidence did not fall one way. Associations reported for children, adolescents or older adults (as classified by the source review) were demarcated.

The sequencing of data extraction allowed for an ‘interpretative trail’ whereby connections presented in the CLD could be readily linked to empirical material in the included reviews [25]. Additionally, it permitted more than one author to follow each stage of data extraction, interpretation and synthesis.

#### Reporting

Although this is not a traditional realist review, reporting was informed by the *RAMESES* publication standards for realist syntheses [28]. These standards are modelled closely on *PRISMA* guidelines but diverge in some requirements to suit the purpose and needs of explanatory reviews (like the current review) compared to reviews summarising the evidence for hypothesised, pre-specified relationships.

### System overview and causal loop diagrams

Systems-based analysis was performed sequentially to identify: system elements, feedback loops, structure, paradigm and goals; the four steps of this analysis are outlined in Table 1. Embedded in systems dynamics theory, this novel analysis plan was developed for the current study and embedded in the theoretical characterisation of complex adaptive systems developed by Donella Meadows [20] and Johnson et al.’s corresponding *Intervention-Level Framework* [29]. Other than positing the role of micro-, meso- and macro-level factors in influencing the accessibility, availability, affordability and acceptability of food, no assumptions about the system were made a-priori: as such building the CLD was first and foremost a data-driven exercise. In brief, elements, connections and feedback loops were directly taken from the findings of our umbrella review to create an initial CLD; this CLD informed how the structure, paradigm and goals were derived by an expert panel and further iterations of the CLD. The CLD was built using STICK-E software (STICK-E version 2,© Deakin University).

**Table 1:** CLD development and analysis

Step; process; CLD iteration	Sequential method of CLD development and analysis	Identified system characteristic [20, 29]
Step 1: setting system boundaries Top-down conceptualisation; prior to iteration	The *boundaries* of the system were determined by the literature review examining socioecological determinants of dietary intake, assuming a conceptual model whereby individual, social, physical environmental, economic and political determinants drive food intake.	*Boundaries:* the scope of the system under study.
Step 2: identifying elements, feedback loops and structure Data-driven development; first iteration (whole-system CLD)	Using the data matrix, variables that were reported to have an association with dietary intake and determinants of dietary intake were entered as *elements* of the CLD. Connections between elements were drawn based on reported presence, direction and polarity (positive, negative) of associations between elements. *Feedback loops* were identified only where drawn connections revealed a ‘closed’ loop, i.e. element A ➔ element B ➔ element C ➔ element A. This process to develop the initial CLD was led by AS and reviewed by KS and FvL.	*Elements*: included variables *Feedback loops*: connections between elements in reinforcing loops denoting growth/decline and balancing loops denoting stabilisation. *Structure*: clustering of connections between elements
Step 3: identifying paradigm and structure Top-down review; second iteration (whole-system CLD)	An expert panel was formed of 12 authors and two additional researchers, with expertise in public health nutrition and systems thinking, who communicated via written comments, two face-to-face sessions and one video discussion; not all group members took part in each session. Each member of the review panel was presented with the CLD and provided with an in-depth explanation how the findings from the umbrella review were used to develop the system elements and connections. First, the accuracy and saturation of the CLD was assessed. Accuracy (i.e. exclusion of errors emerging from data extraction and synthesis and verification of of relationships in the CLD as being interpreted from the umbrella review) was tested by reviewing individual connections between elements. Saturation (i.e. completeness in reflecting the evidence base [30, 31]) was assessed using expert knowledge from the expert panel of the evidence base, to overcome potential limitations of the literature search. Additional literature could be integrated at this stage if saturation was not apparent and there was available literature of sufficient quality. Iterations were only made if they were substantiated by empirical evidence. Second, discussion of feedback loops and structure identified in step 2 elucidated key mechanisms operating within the system. These mechanisms were discussed in relation to four dimensions of the food environment, to explicate the perceived arrangement (temporal ordering and interaction) of mechanisms pertaining to accessibility, availability, affordability and acceptability. This exercise enabled the articulation of the *paradigm* and *structure* of sub-systems. Elements that were present in multiple sub-systems were manually highlighted as ‘link elements’, to demonstrate interconnection between sub-systems.	*Paradigm*: system’s deepest held beliefs derived from goals and sub-system structure. *Structure*: sub-systems derived from feedback loops and connections between elements.
Step 4: identifying goals of sub-systems Top-down conceptualisation; final iteration (overview, sub-system CLDs)	Following the organisation of sub-system CLDs and articulation of the system paradigm within the system overview, the *goal* of each sub-system was derived from feedback loops and sub-system structure.	*Goals*: aims of the system conforming to the system’s paradigm, derived from feedback loops and sub-system structure.

## Results

Across 43 included reviews, 8 studied adults, 2 studied older adults, 13 studied children and adolescents and 20 studied populations across age groups (Table 2). Three reviews exclusively targeted low-income (or proxy) groups; the remaining 40 reviews examined low-income group either in a section of the review or by examining determinants across groups. Twenty of the 43 included reviews were non-systematic reviews.

**Table 2:** Included review characteristics

First author, year	Review focus	Review type; number of relevant studies	Age group and further characteristics^a^	Country / region^a^	Dietary outcome^a^	Income-related outcome^a^
Akande, 2015 [32]	Dietary consumption patterns and physical activity	Systematic; quantitative, qualitative; k = 9	Adults (Canadian Inuit)	Canada	Consumption patterns; access to healthy food	Socioeconomic factors (income and education)
Attree, 2005 [17]	Diet and nutrition in low-income families	Systematic; qualitative; k = 11	Female adults (mothers); low-income	UK	Diet and nutrition	Income
Attree, 2006 [33]	Diet and nutrition issues in UK policies affecting low-income households	Systematic; qualitative; k = 32	All; low-income	UK	Diet and nutrition	Income
Ball, 2006 [34]	SES and obesity	Non-systematic; k = 11	All	HUMIC	Dietary intake	SES
Bomberg, 2017 [35]	Obesity-related behaviours in adults, children and pets	Non-systematic; k = 10	Children; adults	Not specified	Numerous	SES; area deprivation; poverty
Bonaccio, 2016 [36]	Socioeconomic determinants of Mediterranean diet	Non-systematic; k = 9	Adults	HUMIC	Dietary pattern	SES
Correa, 2015 [10]	Built environments correlates of obesity	Non-systematic; quantitative; k = 7	Children; adolescents	HUMIC	Dietary intake	Neighbourhood income
Darmon, 2008 [6]	SES differences in diet quality and causal mechanisms	Non-systematic; k = 51	All	HUMIC	Diet quality (consumption; variety)	SES
Darmon, 2015 [14]	Food prices and socioeconomic disparities in diet quality	Systematic; k = 16	All	Not specified	Diet quality	Income
De Ridder, 2017 [37]	Health impact, prevalence, correlates and interventions for healthy diets	Systematic; k = 12	Adults	HUMIC	Pattern of food consumption	SES (education, work status, income)
Di Noia, 2014 [38]	Determinants of F&V intake	Systematic; quantitative; k = 58	Children; adolescents	HUMIC	Intake (F&V)	Income
Dowler, 2008 [39]	Policy responses for nutritional needs in low-income households	Non-systematic; k = 29	Households	UK	Dietary pattern	Income
Doyle, 2017 [40]	Determinants of (changing?) dietary patterns and quality during pregnancy	Systematic; quantitative; k = 3	Female adults (pregnant)	HUMIC	Diet quality	Income
Dunneram, 2015 [41]	Determinants of eating habits among older adults	Non-systematic; quantitative, qualitative; k = 3	Older adults	Global	Diet quality	SES
Hanson,2014 [42]	Food insecurity and dietary quality in adults and children	Systematic; quantitative; k = 26	Children; adults	USA	Diet quality	Income
Hartmann-Boyce, 2018 [43]	Effectiveness of grocery store interventions on purchasing behaviour and consumption	Systematic; quantitative; k = 12 low-SES groups; k = 6 analysis by SES	Children; adults	HUMIC	Purchasing behaviour	SES (household income; area income)
Hawkes, 2015 [44]	Policies for obesity prevention	Non-systematic; k = 21	All	Not specified	Dietary consumption	SES
Janssen, 2017 [11]	Determinants of out-of-home foods	Non-systematic; k = 21	All	HUMIC	Dietary consumption	Area deprivation, SES group
Krolner, 2011 [45]	Determinants of fruit and vegetable consumption	Systematic; qualitative; k = 6	Children; adolescents	HUMIC	F&V consumption	SES; household income
Lawman, 2012 [46]	Family and environmental correlates of health behaviours	Non-systematic; quantitative; k = 12	Children; adolescents (10–18 years; risk of metabolic disorder due to sociodemographic factors)	Not specified	Dietary intake (F&V; fat; nutrients; fast food)	SES
Leech, 2014 [47]	Clustering of diet, PA and SB	Non-systematic; quantitative; k = 18	Children; adolescents	HUMIC	Diet quality	SES
Lovasi, 2009 [48]	Built environments and obesity	Systematic; quantitative; k = 4	All	USA	Dietary intake / Access to healthy foods	Disadvantaged populations
Mcphie, 2014 [49]	Correlates of maternal child feeding practices	Systematic; quantitative; k = 7	Children (2–6 years)	HMIC	Feeding practices	Maternal education or household income
Minaker, 2016 [50]	Retail food environments	Scoping; quantitative, qualitative; k = 28	All	Canada	Access to healthy food (food choice)	Area SES
Myers, 2018 [51]	Food craving and body weight	Non-systematic; k = 2	Adults	Not specified	Food cravings	Income, food insecure groups, disadvantaged groups
Nicklett, 2013 [12]	F&V intake in older adults	Scoping; quantitative, qualitative; k = 13	Older adults	Not specified	F&V purchases and intake	Household income; neighbourhood deprivation
Ohly, 2017 [52]	Low-income pregnant women and Healthy Start programme	Realist; k = 38	Female adults (pregnant)	UK, USA	Purchasing decisions	Income
Olstad, 2017 [16]	Targeted obesity policies for obesity-related behaviours in low SES populations	Systematic; quantitative; k = 18	Children; adults; low-SES	HUMIC	Dietary intake	SES
Ontai, 2009 [53]	Family-based obesity prevention in low-income children	Non-systematic; k = 2	Children	Not specified	Dietary intake,	Income
Oostindjer, 2017 [54]	School meals and diet	Non-systematic; k = 12	Children	Global	Intake and dietary behaviour	Income
Osei-Kwasi, 2016 [55]	Determinants dietary behaviour in ethnic minorities	Systematic mapping; quantitative, qualitative; k = 37	All	Europe	Dietary behaviour	Ethnic minority groups with low income
Pampel, 2010 [56]	Socioeconomic disparities in health behaviours	Non-systematic; quantitative, qualitative; k = 13	All	Not specified	Diet	SES
Paquette, 2005 [57]	Perceptions of healthy eating	Non-systematic; quantitative, qualitative k = 2	All	Global	Perception of health Intake	SES
Pitt, 2017 [58]	Local food environments and food behaviours	Systematic, meta-analysis; qualitative; k = 30	Adults	HUMIC	Food behaviour	SES
Power, 2005 [59]	Determinants of healthy eating in low-income Canadians	Non-systematic; k = 69	All	HUMIC	Eating behaviour	Income
Robinson, 2008 [60]	F&V intake in low-income African Americans	Systematic; k = 13	All	USA	F&V intake	Income
Robinson, 2012 [61]	Development of obesity in infancy and children	Non-systematic; k = 9	Infants; children	Not specified	Consumption; purchasing	Household Income, SES
Savage, 2007 [62]	Parental influence on eating behaviour	Non-systematic; k = 2	Infants; children; adolescents	Not specified	Intake; eating behaviour	SES, income
Scaglioni, 2018 [63]	Factors influencing children’s eating behaviours	Systematic; k = 3	Infants; children; adolescents	HUMIC	Eating behaviours	Income, education, SES, employment, area advantage
Shemilt, 2013 [64]	Economic instruments for population diet and PA behaviour change	Systematic scoping; quantitative; k = 65	All	Global	Intake; behaviour; purchasing	Income
Sigman-Grant, 2015 [65]	Family resiliency and obesity in young children	Non-systematic; k = 6	Children	Not specified	Dietary intake	Household income, neighbourhood income
Story, 2008 [13]	Policy and environmental approaches to healthy food and eating environments	Non-systematic; k = 9	All	USA	Access to healthy food	Neighbourhood income
Zarnowiecki, 2014 [66]	SES and predictors of children’s dietary intake	Systematic; quantitative; k = 28	Children (9–13 years)	HUMIC	Dietary intake	SES

^a^Scope of the review, rather than relevant studies considered for data extraction. *HUMIC* higher- or upper-middle-income country, *SES* socioeconomic status

Seventy-three dietary determinants were extracted; 45 determinants were categorised using the *DONE* framework (Additional file 1); 28 determinants specific to low-income groups could not be sufficiently categorised using existing *DONE* variables and were captured using additional variables within *DONE* categories. Thirteen determinants were not included in the system map either because, on balance, the literature reported a non-significant association, or as demographic variables, there were treated as proxies for exposures rather than causal determinants (for example, ethnicity was considered an exposure for residential segregation and residential segregation was considered the causal determinant for accessibility of food, rather than ethnicity).

### System paradigm and sub-system structure

**Fig. 2:**
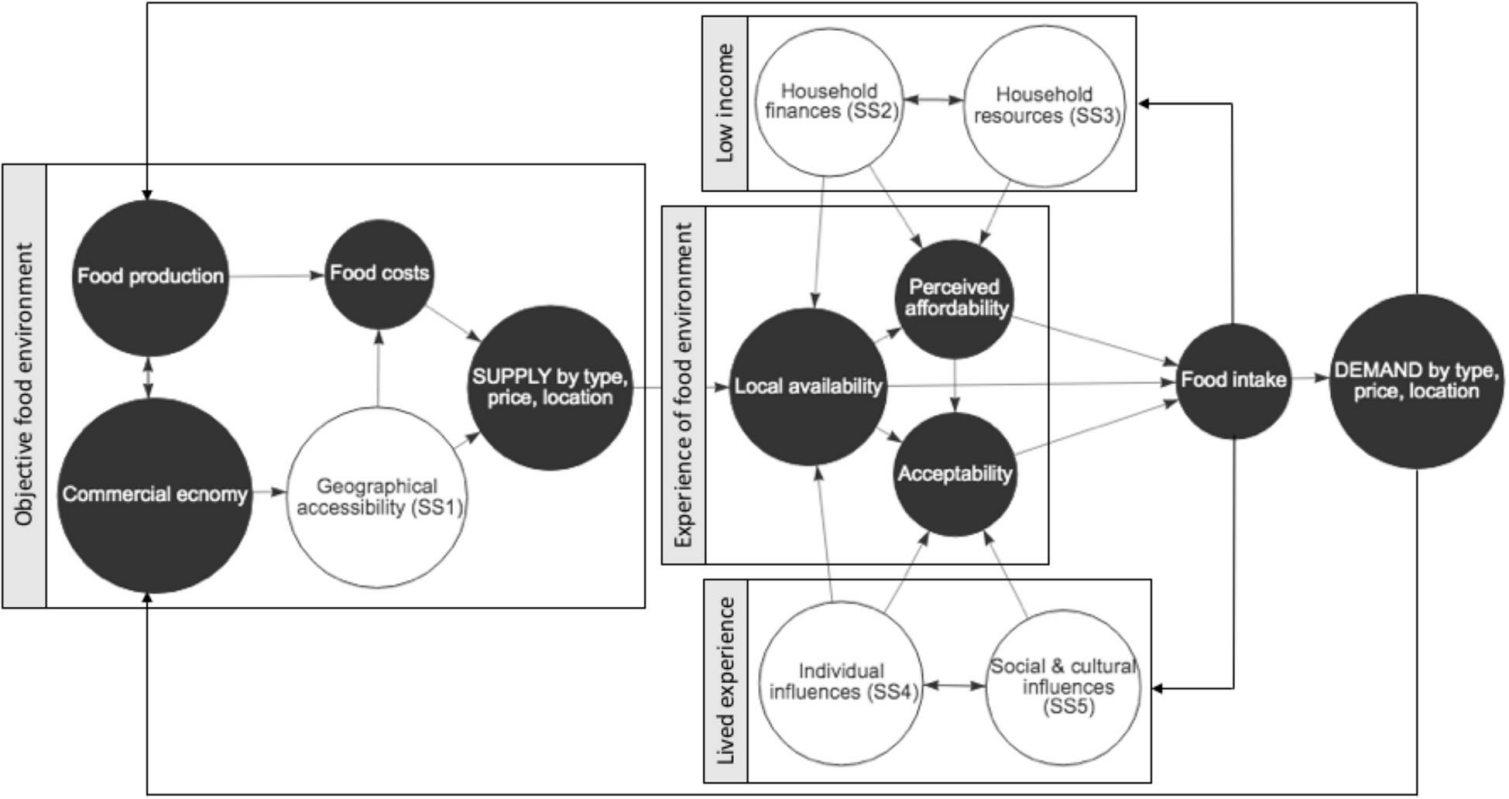
Overview of system driving food intake in low-income groups

**Fig. 3:**
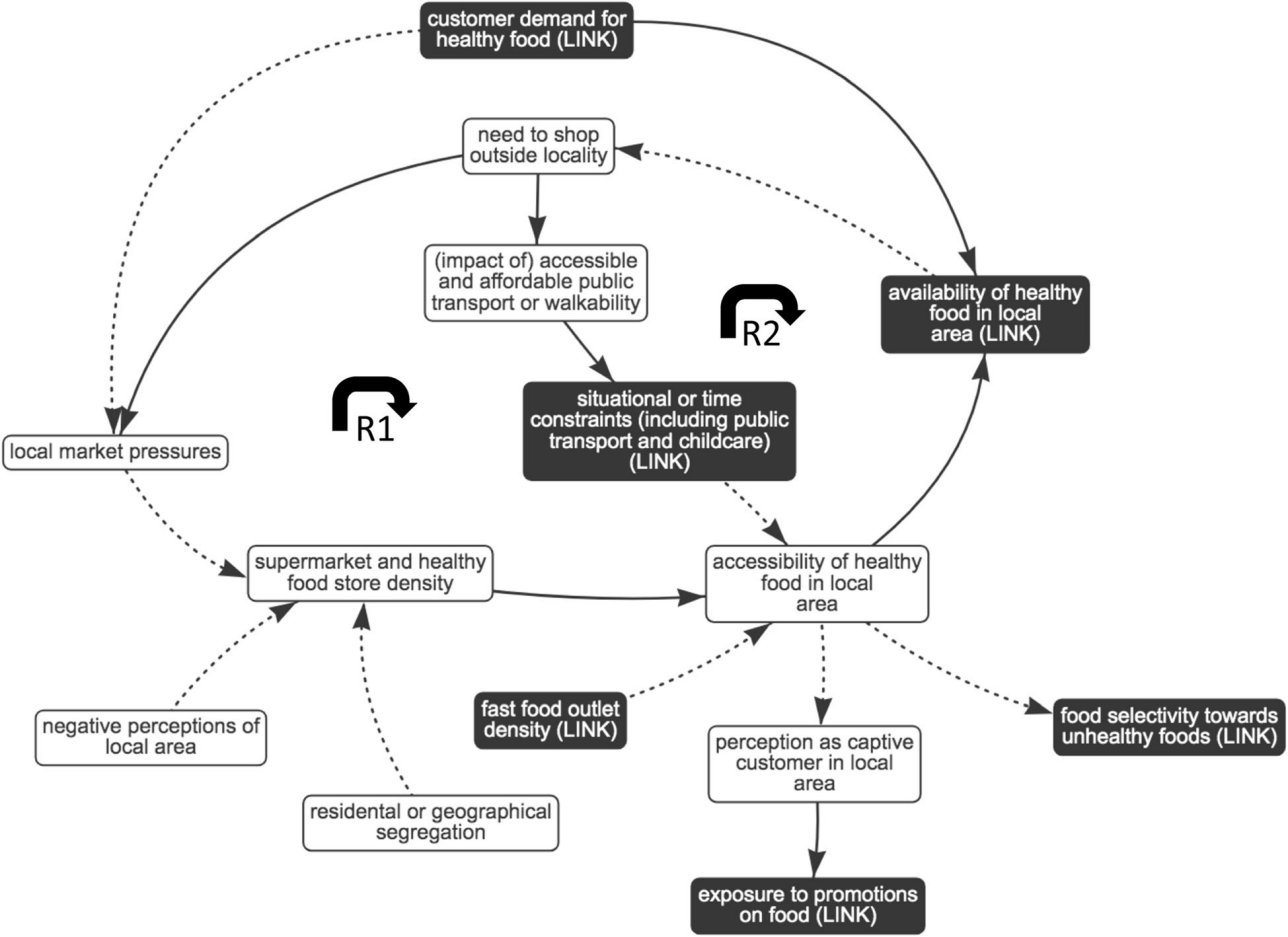
Sub-system 1: geographical access. Dotted arrows indicate a negative association; solid arrows indicate a positive association; black box indicates presence in other sub-system, i.e. ‘link’ element; R1 and R2 indicate reinforcing feedback loops

**Fig. 4:**
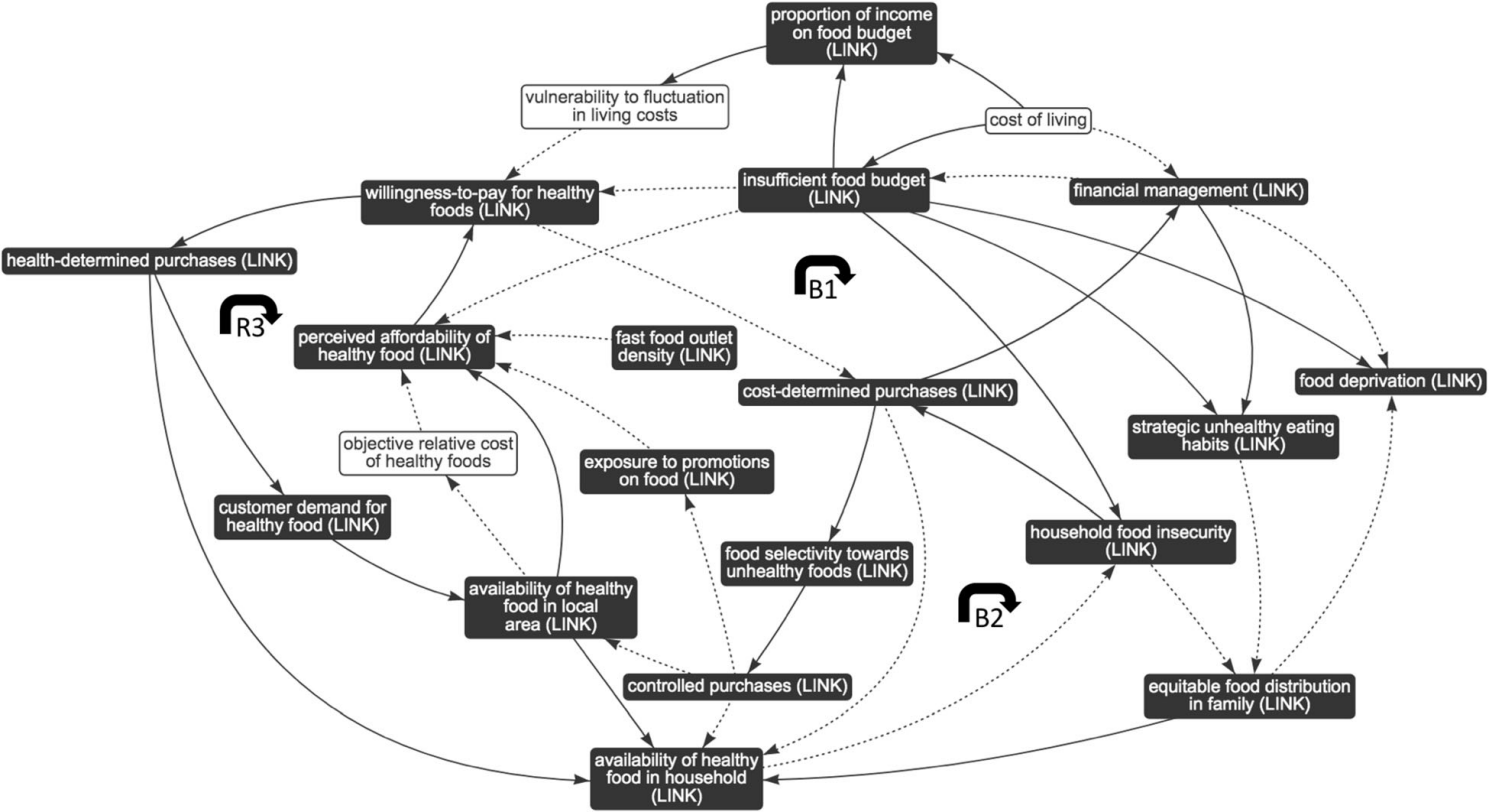
Sub-system 2: household finances. Dotted arrows indicate a negative association; solid arrows indicate a positive association; black box indicates presence in other sub-system, i.e. ‘link’ element; B1 and B2 indicate balancing feedback loops; R3 indicates reinforcing feedback loop

**Fig. 5:**
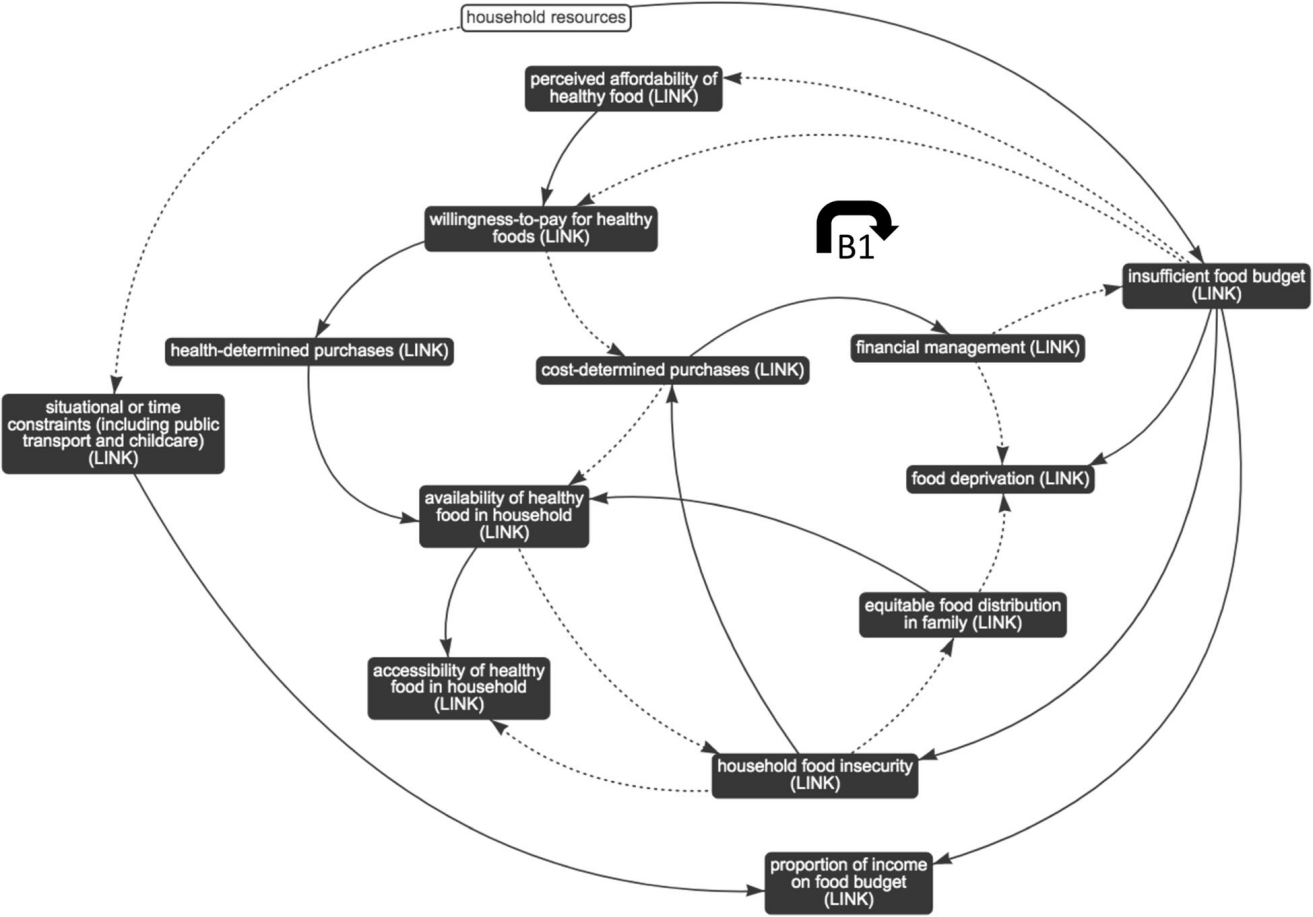
Sub-system 3: household resources. Dotted arrows indicate a negative association; solid arrows indicate a positive association; black box indicates presence in other sub-system, i.e. ‘link’ element; B1 indicates balancing feedback loop

**Fig. 6:**
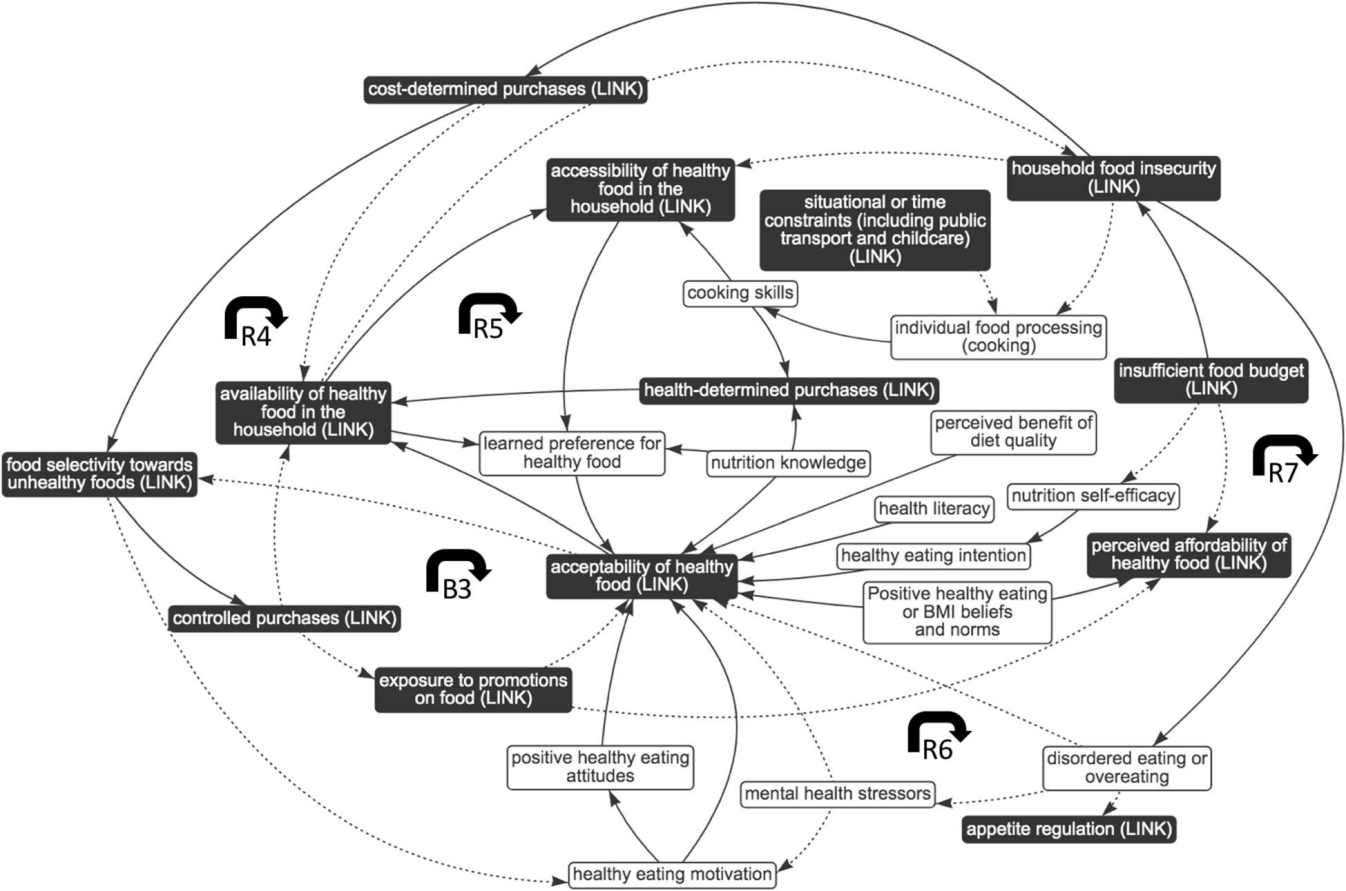
Sub-system 4: individual influences. Dotted arrows indicate a negative association; solid arrows indicate a positive association; black box indicates presence in other sub-system, i.e. ‘link’ element; B3 indicates balancing feedback loop; R4–7 indicate reinforcing feedback loops

**Fig. 7:**
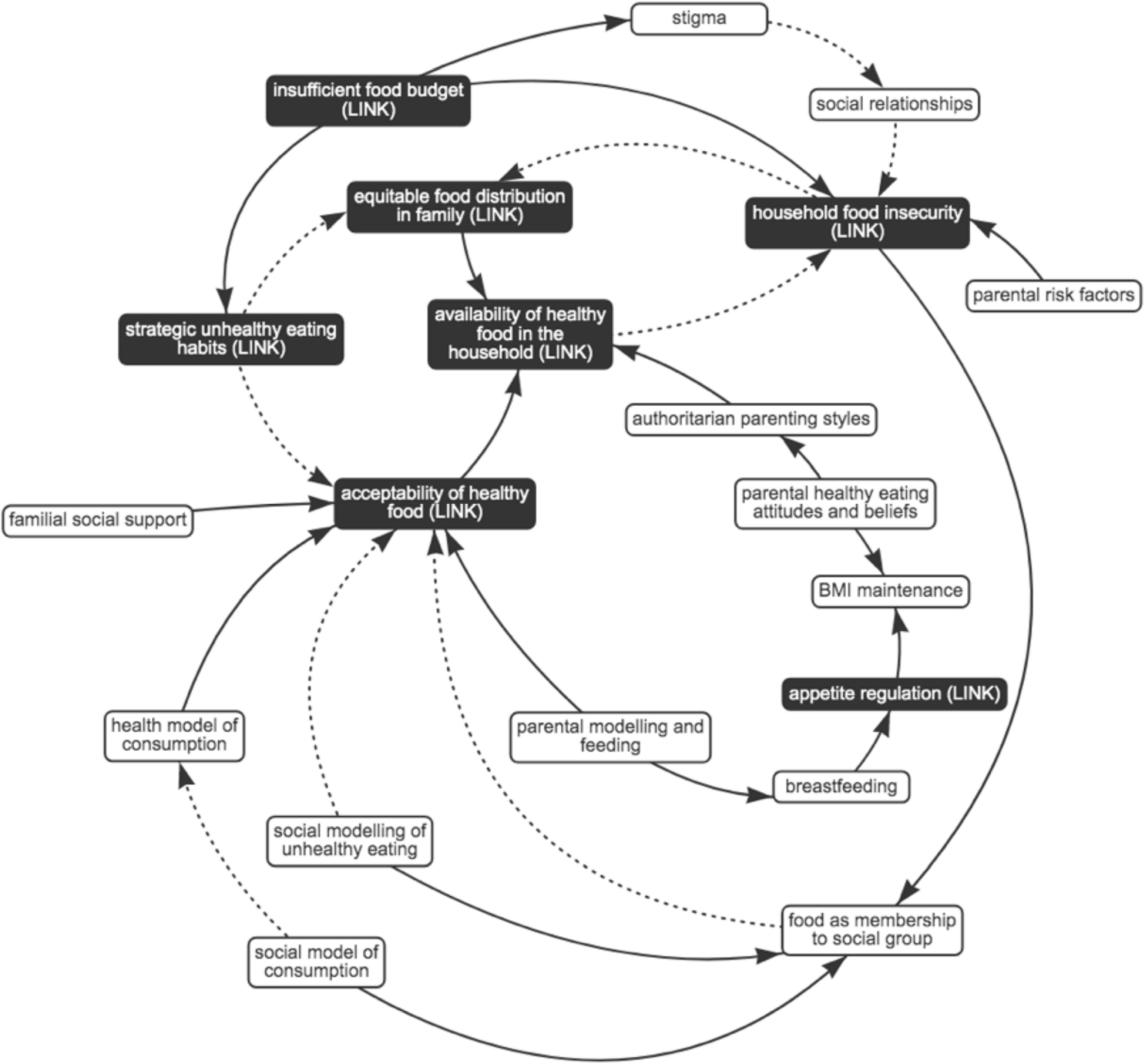
Sub-system 5: social and cultural influences. Dotted arrows indicate a negative association; solid arrows indicate a positive association; black box indicates presence in other sub-system, i.e. ‘link’ element; R8 indicates reinforcing feedback loop

The steps outlined in Table 1 led to the articulation of a wider system comprising five sub-systems. The overview of the system and the arrangement of sub-systems (SS1–5) is presented in Fig. 2. In support of our proposal, it was possible to observe complex adaptive sub-systems which influence the accessibility, availability, affordability and acceptability of healthy food in low-income groups. Individual sub-systems (titled: ‘geographical accessibility’, ‘household finances’, ‘household resources’, ‘individual influences’ and ‘social and cultural influences’) are presented as CLDs (Figs. 3, 4, 5, 6 and 7) comprising elements (i.e. variables), connections and feedback loops that were identified in step 2 (Table 1).

Interaction between sub-systems was observed when the same elements or feedback loops appeared in multiple sub-systems. This enabled the arrangement of sub-systems around the dimensions of the food environment (Fig. 2) and thereby enabled the conceptualisation of the system paradigm as a supply-and-demand loop operating in an economic paradigm, with the need for economic prosperity as the system’s deepest held belief. On the supply end: food production and commercial economy can be assumed to influence the cost and local accessibility of food types. This shapes local community and consumer availability of food, which together with income and lived experience, contributes to perceived, or realised, affordability and acceptability of dietary patterns, behaviours and intake. Together, availability, affordability and acceptability is thought to determine propensity for healthy versus unhealthy dietary intake, which culminates over time in the demand for food type, prices and geographical provision. This feeds back to inform the supply of food and so on. In essence: over time, low-income individuals’ experience of the food environment is shaped by the economic market and this market is shaped by the demand generated by individual experience of the food environment when faced with the constraints of a low income. The number of ‘link’ elements in the sub-systems (Figs. 3, 4, 5, 6 and 7) indicates a high degree of interconnection between sub-systems.

### Sub-systems, goals and feedback loops

Sub-system CLDs presented in Figs. 3, 4, 5, 6 and 7 depict evidence-based connections between elements, but do not aim to depict the strength or size of the evidence base. Following conventions for CLD development, where possible, elements are phrased in a neutral way. Reinforcing (R; denoting growth or decline) or balancing (B; denoting stabilisation) feedback loops within sub-systems were used to identify system goals (as outlined in step 4, Table 1). Additional relationships presented between elements which are not a part of feedback loops are not discussed in the interest of space, but do warrant acknowledgement as: i. evidence-based relationships contributing to dietary intake in low-income groups, and ii. opportunities for individual heterogeneity or setting the conditions for the feedback loop.

#### Sub-system 1: interplay between the food environment and geographical access

The dynamics of this sub-system are relevant to low-income households in low-income neighbourhoods. Lower density of supermarkets and healthy food outlets and higher density of fast food outlets are reported in low-income neighbourhoods [10, 11, 13, 14, 34, 35, 44, 50, 56, 61]. Placement of this sub-system in the wider system suggests that this is a result of a long-term supply-and-demand feedback loop, whereby the multiple influences on demand lead to differential geographical distribution of the food supply. Feedback loops within the sub-system provide insights into why this situation may be reinforced over time.

As shown in reinforcing feedback loop 1 (Fig. 3, R1), research predominantly shows that in low-income neighbourhoods, healthy food is less accessible and the reduced local availability encourages residents to shop outside of the local area for these products [39, 48, 58]. As a result, there may be increased economic pressure on the few healthy food outlets in the neighbourhood, who compete for remaining customers [12, 48, 58]. It is reported that these healthy outlets are more likely to start stocking less healthy food, increase the price of healthy food in order to make profit or close due to lack of trade [12, 48, 58, 61]. This reduction in local availability can further increase residents’ need to shop outside their locality [58].

Moreover, illustrated in reinforcing feedback loop 2 (Fig. 3, R2), evidence suggests that residents in low-income neighbourhoods more often need to shop outside their locality but have limited access to private vehicles and rely heavily on public transport [14, 33, 58, 60]. Where transport provision is poor and levels of walkability are low, public transport can be costly and unreliable but may be the only available option [39, 58, 60]. These impediments are compounded by accompanying children if childcare is not easily or freely available [17, 58], increasing the cost of transport and decreasing the ease of walking long distances, which further reduces realised accessibility and availability. This influences purchasing frequency and decisions (e.g. avoiding perishable and heavy items such as vegetables or grains) and can increase residents’ exposure to the marketing and trading techniques which are reported to be employed by local outlets to increase profit (e.g. promotions on attractive unhealthy items, poorer quality fresh produce as the shop is unable to frequently rotate stock) – a circumstance described by residents as being a ‘captive customer’ [36, 58]. In conclusion, the distribution of access across different neighbourhoods can be reinforced over time. The situation at an individual level may be compounded by customers’ reliance on inadequate public transport system and low neighbourhood walkability.

From the reinforcing and balancing feedback loops, the authors observed the goal of this sub-system as the economic growth of larger outlets which have a trading advantage, and the commercial efficiency or survival of smaller, local outlets.

#### Sub-systems 2 and 3: interplay between the food environment and household finances and resources

Evidence suggests that energy-dense, low-nutrient foods (comprising refined grains, added sugars and saturated fats) are often cheaper to purchase (on a cost-per-calorie basis) than healthier, fresh produce such as fruits and vegetables [6, 11–14, 36, 37, 44, 56, 61]. Shown in balancing feedback loop 1 (Fig. 4, B1), insufficient budgets for food can drive purchasing decisions which prioritise cost-effectiveness (using multiple metrics to determine ‘true cost’, or value) [37, 45, 58] and therefore reduce willingness-to-pay for healthier items which may be objectively and/or subjectively more expensive. Cost-determined purchases of cheaper, energy-dense foods may enable financial management of the household and reduce financial strain. It is usually not possible to manage the financial situation to the extent that the food budget is sufficient enough to alleviate the need for cost-determined purchases. Research shows that this strategy can be perceived as effective in the short-term in escaping food deprivation [39]; it is therefore maintained. Cost-determined purchases can lead to the selection of cheaper, unhealthy foods and tightly-controlled purchases forming a monotonous diet with little opportunity for waste, but which contribute to long-term food insecurity, which can lead to irregular consumption of adequate and nutritious food (Fig. 4, B2) [6, 13, 14, 42].

Moreover, presented in reinforcing feedback loop 3 (Fig. 4, R3), limited health-determined purchases may reduce customer demand for healthy produces. This has been shown to impact availability in the local area and increase the cost of stocked items of healthy, fresh produce due to lower stock turnover [12, 39, 50, 55, 58, 60]. In turn, it is reported that individuals perceive healthier, fresh produce as more expensive, which can further reduce the willingness-to-pay for healthier items when prioritising cost-effectiveness [6, 12, 37, 45, 58].

Households with fewer income-related resources report insufficient food budgets: although the absolute cost of food tends to be lower in low-income groups, the proportion of household income spent on food increases [12](Fig. 5). Budgets are often further reduced due to situational and time constraints owing to a lack of household resources (e.g. lack of access to a private vehicle, reduced access to childcare) and vulnerability to fluctuations in living costs (e.g. irregular expenses) [39, 52, 58]. As explained in balancing feedback loop 1 (Fig. 4, B1), individuals are more likely to make cost-determined purchases (rather than health-determined purchases), in order to manage their financial situation and food budget. Moreover, evidence shows that households with limited resources are at much higher risk of household food insecurity [32, 59], placing individuals at an increased risk of food deprivation, particularly mothers (due to inequitable food distribution in the family, which prioritises children) [17, 59] and those living alone [39].

Sub-systems 2 and 3 were conceived, by review of the feedback loops, as sharing a single goal: the strategic mitigation of limited finances and resources.

#### Sub-system 4: interplay between the food environment and individual influences

In Fig. 6, reinforcing feedback loop 4 (R4) comprises a long causal pathway: household food insecurity may prompt cost-determined purchases that can lead to the selection of relatively cheap, unhealthy foods and reportedly can decrease individuals’ motivation to eat healthily (due to the dominating influence of cost) [6, 33, 58]. Because motivation to eat healthy foods is not supporting the acceptability of healthy foods, availability of healthy food in the household is likely to be reduced, contributing to food insecurity [6, 37]. Relatedly, balancing feedback loop 3 (Fig. 6, B3) shows that food selectivity for unhealthy foods may lead to controlled purchasing (i.e. inflexible selection of food items according to pre-specified plans around food quantity, price and/or type), which is reported to limit individuals’ interest in price promotions on both healthy and unhealthy items [36]. A reduced interest in promotions on healthy items means these items can continue to be perceived as more expensive and, therefore, less acceptable; as a result, cost-determined purchasing strategies still favour unhealthy items [33, 36].

Reinforcing feedback loop 5 (Fig. 6, R5) illustrates how increased exposure to energy-dense, nutrient-poor foods (due to the wider system) can inform learned food preferences [44, 54, 63] and thus heighten acceptability of unhealthy foods over healthier options [38, 66]. It is reported that acceptability informs purchases, in order to avoid the risk of trying new foods (leading to potential waste) on a low food budget [33]. This ensures further exposure which reinforces food preferences.

Food insecurity is another important influence on food acceptability – demonstrated in reinforcing feedback loops 6 and 7 (Fig. 6, R6, R7). Food insecurity comprises periods of food scarcity and relative adequacy that may lead to disordered eating or overeating in order to manage stress, gain perceived periodic control over food choices and reduce the effect of food deprivation (in the individual or dependent children) [33, 51, 59]. As energy-dense, nutrient-poor food is more likely to be perceived as acceptable and provides heightened physiological reward, disordered eating or overeating often involves overconsumption of unhealthy foods [56]. This prompts their availability in the household and thus increases food insecurity as it reduces consistent availability of healthier foods. In addition, loop 6 shows that overeating can be used to manage mental health stressors, further reducing the relative acceptability of healthier foods [35, 56].

The goal of sub-system 4 is conceived, by review of the feedback loops, as achieving acceptable cost-determined and controlled purchasing (i.e. inflexible selection of food items) based upon preferences cultivated by disproportionate exposure to unhealthy foods. This goal is reported to undermine pathways promoting health-determined purchasing (determined by nutrition knowledge and cooking capability [6, 11, 34, 37, 45, 66, 67]), contributing to low perceived self-efficacy, motivation and perceived benefit of healthy eating which are related to diet quality [6, 33, 37, 66]. It is probable that different time scales will reveal further dynamics of this sub-system, for example, mechanisms influencing the changing acceptability and affordability of healthy versus unhealthy foods, over time and across the wider system (Fig. 2).

#### Sub-system 5: interplay between the food environment and social and cultural influences

As presented in Fig. 7, research suggests that health and social models of food consumption (respectively, focusing on the health benefits of food and diet, or the social practices around and cultural meaning of food and diet) can be in competition when they diverge in the types of food they endorse [60]. A social model of consumption is observed as being important in low-income groups, as in higher-income groups [59, 60, 65]. Research describes this in relation to the experience of sharing food as an important aspect of consumption; using food as a marker of good parenting [11, 65]; and signifying social or cultural status by buying specific foods (e.g. branded items, fast food) or visiting certain shops [11, 33, 58]. Shown in reinforcing feedback loop 8 (Fig. 7, R8), in order to gain control over and pleasure in food choices in the face of food insecurity, food can be increasingly viewed as a marker of membership to a social group, which may include other food-insecure households [59]. This can increase the acceptability of foods such as branded snacks, fast food or processed ready-meals [11, 33], in turn increasing their availability and the insecurity of nutritious foods. Together, this can undermine the potential influence of the health model on food consumption [60].

Social networks are reported to be protective against food insecurity in some minority ethnic groups [6], but larger family size can lead to food deprivation as a result of food insecurity [32, 59]. This sub-system is likely to be influenced by a long-term feedback loop across the wider system, which determines the social norms and attitudes underpinning social models of food consumption.

The goal of sub-system five is conceived as achieving alignment with prevailing social and cultural models of food consumption.

## Discussion

Using a novel method, it was possible to systematically synthesise quantitative and qualitative evidence from 43 reviews in a way that elucidated the dynamics of a complex adaptive system of determinants of the unhealthy food environment in low-income groups in higher- and middle-income countries. The system was interpreted as operating within an economic paradigm with a structure of multiple sub-systems working towards goals that create and sustain a food environment that increases the relative accessibility, availability, affordability and acceptability of unhealthy foods compared with healthy foods. An economic basis of the food environment results in ubiquitous supply of energy-dense, nutrient-poor and ultra-processed foods, which fuels demand of these products based on their social and cultural significance, availability and affordability. In light of potential attempts to use the acknowledged complexity of this public health issue to justify inaction [68], it is vital to use evidence-based insights into this complex adaptive system to inform policy and interventions.

The dynamics around ‘cost-determined purchasing’ exemplify the complex pathways through which multiple distal and proximal determinants operate to reinforce an adverse food environment. Low-income households spend a greater proportion of their income on food, despite spending less in absolute terms. For example, in France, households in the top and bottom deciles of income spent 22% and 29% of their disposable income on food, respectively [69]. As demonstrated in feedback loop B1, food cost is of paramount importance to low-income groups, but cost, or value, is not determined by price alone [18]. Evidence from our umbrella review indicates that unhealthy foods tends to be selected in cost-determined purchases due to objective and relative characteristics of the product (e.g. price, longevity, palatability, brand-allegiance, potential for satiety), restricted financial ‘slack’ prohibiting long-term planning (e.g. bulk-buying, promotions, store-cupboard ingredients, cooking from scratch), individual capability (e.g. cooking skills) and acceptability in the household (e.g. avoidance of food waste). Consequential prioritisation of unhealthy foods in cost-determined purchasing (feedback loops B1–2, R3–4) pushes sociocultural and individual dynamics to favour unhealthy food intake (feedback loops B3, R5, R7), which over time reinforces the acceptability and affordability of these foods (due to the wider supply-and-demand loop), further strengthening their favour in cost-determined purchases. This vicious cycle perpetuates the economic, social, cultural and individual conditions for the intake of unhealthy foods, even though the system goals of household economic, sociocultural and individual sub-systems are not, intrinsically, meant to encourage unhealthy food intake (rather, they are conceived as: mitigation of limited finances and resources and alignment with learned preferences and social models of food intake). Arguably the only parts of the system intrinsically linked to unhealthy food intake are concerned with the economics of the production and supply of unhealthy foods.

From the observation of these dynamics, it follows that it is unlikely that a sustainable effect on dietary intake will be achieved through isolated interventions aiming to increase the accessibility, availability, affordability or acceptability of healthier products. For example, a new supermarket in a low-income neighbourhood may do little to change purchasing habits [16]. In order to achieve impact, systems-based analysis can be used to identify entry or leverage points in the system, where interventions can have a larger impact by acting on important mechanisms (e.g. reinforcing feedback loops) that are driving system outcomes. Such analysis could identify interventions that might have desirable knock-on effects on other parts of the system or demonstrate how multiple interventions could work in synergy to foster favourable changes in system functioning.

Our analysis suggests that in order to have most impact on the mechanisms driving this interconnected system, interventions could either use structural actions (that do not call on personal agency) to circumvent social, cultural and individual dynamics underlying dietary intake (such as encouraging reformulation of food composition through taxation or bans) or modify the dynamics steering cost-determined purchases towards unhealthy foods. For example, economic interventions aiming to facilitate longer-term financial management in low-income households may increase the perceived affordability of healthier foods which can be bought in bulk to comprise a ‘stock-cupboard’ which permits cooking from scratch. Simultaneously, ideas such as ‘bulk buying clubs’ could be investigated as a way to distribute the cost or perceived risk of bulk items across small community groups; research should examine how such ‘clubs’ can be accessible and affordable for low-income groups [70, 71].

Interventions targeting the dynamics underlying the acceptability of healthy foods (sub-systems 4 and 5) could also be effective in modifying cost-determined purchases. Evaluations of school-based free fruit schemes have reported little effect on overall energy intake or fruit intake outside of school [16]. This may be in part because the intervention might not foster broader acceptability of fruit, so parents still view fruit as a relatively costly food item (which is now provided by the school) compared with branded snacks that are more acceptable as they signify social or cultural status [11, 33]. Incorporating a social aspect to such interventions could reinforce the acceptability of healthy food items and bridge eating behaviour in school and at home. Community food resources such as urban gardens and orchards or school chicken coops, could provide opportunities to modify perceptions, create social practices and increase exposure to healthy, fresh foods [72]. Co-design of interventions or implementation strategies also provides a way to engage target populations – such as school children and their parents - and reduce unintended and unexpected consequences of interventions, such as increased use of competing local food outlets in response to healthier food offerings at school canteens [73]. Finally, structural actions could also be useful; for example, subsiding rent for outlets which predominantly sell fresh, unprocessed, nutrient-rich foods (e.g. salad bars, health food shops). This may allow healthy outlets to compete with unhealthy takeaways that often serve a social function and, as demonstrated in feedback loop R1, tend to possess a competitive advantage allowing them strategic locations near residential areas, schools and leisure facilities. Analysis of the system alongside data from the target population should allow the prioritisation of actions according to context and suggest which actions need to be implemented simultaneously to achieve impact.

System dynamics theory posits that feedback loops can suppress or potentiate the effect of policy [74, 75] and that complex systems are adept at continual self-organisation, to resist modification [20, 21]. The number of feedback loops and the structure of the observed system across multiple sub-systems, suggest that the effects of any intervention may manifest over considerable time or in surprising ways, or may be suppressed by compensatory adaptation elsewhere in the system [76]. It is therefore likely that entrenched economic, social and cultural practices around food, that are produced by long-term feedback loops across the wider system, will only change as a result of a paradigm shift which promotes healthy dietary intake rather than economic prosperity [77, 78]. Without change at the highest level of the system, even policies which promise a desirable health and equity effect, such as increased taxation on sugar-sweetened beverages, are likely to be impeded in their impact. This is because the supply side of the food environment will continue to engineer demand for unhealthy food and drink in low-income groups, even at increased cost to the individual [79]. Directed efforts to prioritise public health across different sectors following the COVID-19 pandemic (e.g. https://www.who.int/news/item/06-04-2021-who-urges-countries-to-build-a-fairer-healthier-world-post-covid-19) may be well placed to engender change at the higher levels of the system. An urgent focus on the commercial determinants of health is warranted in research and practice [80, 81] and future research should aim to further articulate the ‘supply’ side of the system, potentially by using the method outlined in this review.

The current study developed a novel, systematic method of searching the literature, extracting data and synthesising evidence to observe a complex system; the linkage between the reviewed literature and resultant systems map allows new evidence to be integrated [26, 82]. Our results imply that future research in the food environment has no option but to acknowledge a systems perspective when examining the different conditions under which individuals in low-income groups are more or less able to eat healthily. Importantly, while the observed dynamics are thought to shape the food environment, individuals in low-income groups will have differential exposures and vulnerability to that food environment [6, 19]. For example, low-income groups in less disadvantaged neighbourhoods may be exposed to different aspects of the food environment compared to individuals living in more disadvantaged neighbourhoods or individuals living in different cities or countries. It is therefore important to distinguish food environment influences on low-income households and households in low-income neighbourhoods. Similarly, the food environment may be differently experienced by groups characterised by lower levels of education, employment or socioeconomic status (used as proxies for income in this study).

There are also likely to be meaningful variations in the systems determining food environments across countries and regions. The generic systems map developed in this study can be used as a framework to establish system boundaries for more specific CLDs. Additionally, it can prioritise research questions for future systems-based literature reviews and generate specific hypotheses for empirical analyses, both of which can calibrate parts of this generic systems map [26]. As more research becomes available, increased specificity can be achieved. Nevertheless, a cohesive view incorporating generic, global forces (such as the commercial determinants of health) and local, specific mechanisms (such as lived experiences) remains valuable for attempts to change the system structure, goals or paradigm.

The study design has inherent strengths and limitations. As the system boundaries transcend traditional boundaries of public health research, it was necessary to judiciously review and extract data from a wide range of review types. This is a strength in capturing potential dynamics, enhancing the validity of the conceptual model. However, there is unlikely to be a finite set of relevant literature reviews that can be feasibly found through a set of search terms and the defined inclusion and exclusion criteria used within a single umbrella review [25]. In the current review, the search terms were broad rather than specific and no search terms specifically pertained to determinants at the environmental level. Our conceptualisation of the food environment as comprising four key dimensions (accessibility, availability, affordability and acceptability) might have further emphasised a focus on individual-level determinants over environmental or more upstream determinants. To protect against incompleteness, the expert panel was asked to supplement the literature overview, if necessary, in order to achieve saturation of included literature. Future reviews may also include terms such as ‘contextual factor’ to ensure that reviews examining environmental-level determinants are identified. It should be noted that by studying low-income groups, the review may have missed evidence on dietary determinants which have only been examined in the general population and not in low-income groups as there is no theoretical reason to expect differences in the importance of these determinants by income group. Evidence from the general population could supplement findings where appropriate [37, 83, 84].

Similarly, it should be acknowledged that the authors made a-priori assumptions about the principal dimensions of the food environment, following well-established theories of the food environment. While these assumptions will have influenced how we derived and labelled the goals of the system, we purposefully used broad search terms and the *DONE* framework (which expands beyond food environment determinants) for the categorisation of determinants, in a concerted effort to identify the most important determinants of dietary intake.

Finally, it is important to note that evidence underpinning the systems map is drawn in some instances from cross-sectional studies, which cannot determine direction of causality. Following the conventions of using causal loop diagramming as a tool, such associations are presented as assumed causal associations (although it should be noted that often qualitative and longitudinal or experimental evidence substantiated these associations). Using a higher threshold of evidence to support causality is arguably not possible when drawing on an evidence base that has only recently started to adopt complex systems approaches, as the examination of long causal pathways using traditional epidemiology methods is limited. Instead, the systems map draws on evidence, interpretations and assumptions of causality in high-quality quantitative and qualitative literature. It should also be noted that potential publication bias towards significant findings over non-significant findings could have shaped our systems map as could have the inclusion of non-systematic reviews; interpretation of the systems map should acknowledge these potential sources of bias. New evidence should be integrated into the systems map as it becomes available, especially as research pays greater attention to intervening and distal variables influencing diet. Nonetheless, considering the observed inequalities in dietary intake and the demonstrably adverse food environment currently faced by low-income groups, action based on incomplete evidence may be required [16].

## Conclusions

Multiple interconnected feedback loops shape an adverse food environment that increases the accessibility, availability, affordability and acceptability of unhealthy foods, leading to poorer dietary intake in low-income groups. These dynamics were interpreted as emerging from a paradigm supporting economic growth rather than health. Systems-based interventions aiming to improve the food environment and reduce inequalities in obesity-related health outcomes will need to be ambitious, long-term and responsive to unexpected adaptation in the system. In order to be successful, interventions are likely to have to simultaneously target multiple feedback loops in the system – creating the conditions where cost-determined purchases will align with both health objectives and sociocultural and individual preferences. Systems-based research must be a cornerstone of a shared endeavour to reshape the food environment and reduce the burden of disease in low-income groups [76, 78].

## Supplementary Information

**Additional file 1.**

## Data Availability

The dataset derived from the systematic umbrella review are available from the corresponding author on reasonable request.

## Abbreviations

CLD: Causal loop diagram
DONE framework: Determinants of Nutrition and Eating framework
NCDs: Non-communicable diseases
