# Supplementary material for: Dynamics of the complex food environment underlying dietary intake in low-income groups: a systems map of associations extracted from a systematic umbrella literature review
Source: Int J Behav Nutr Phys Act. 2021 Jul 13;18:96. doi: 10.1186/s12966-021-01164-1 (PMC8276221; doi:10.1186/s12966-021-01164-1)
Supplement: Supplementary file 1 — Additional file 1. [file 12966_2021_1164_MOESM1_ESM.docx]

**Additional file 1**

| ***DONE* category** https://www.uni-konstanz.de/DONE/view-interactive-data/ | **CLD element** | ***DONE* variable** |
| --- | --- | --- |
| **Biological (Individual)** | | |
| Food related physiology | appetite regulation | appetite |
| Anthropometrics | BMI maintenance | BMI |
| Sensory perception | learned food preferences | food liking |
| **Demographic (Individual)** | | |
| Biological demographics | age, breastfeeding; gender^✝^ | age; having been breastfed; gender |
| Cultural characteristics | minority ethnicity or race^✝^ | migration background |
| Situational demographics | living alone* | living alone |
| Personal socioeconomic status | personal socioeconomic status^✝^; financial management (addition); cost of living (addition); vulnerability to fluctuation in living costs (addition) | socioeconomic status |
| **Psychological (Individual)** | | |
| Mood and emotions | mental health stressors | negative emotions; depressive symptomatology |
| Self-regulation | self-regulation skills* | self-regulation skills |
| Health cognitions | healthy eating motivation; healthy eating intention; positive healthy eating attitudes (addition); healthy eating or BMI beliefs and norms (addition) | healthy eating motivation; healthy eating intention |
| Food knowledge, skills, abilities | nutrition knowledge; cooking skills; nutrition self-efficacy; health literacy (addition) | nutrition knowledge; cooking skills; nutrition self-efficacy |
| Food beliefs | perceived benefit of diet quality | perceived benefit of diet quality |
| Food habits | food habits* | food habits |
| Eating regulation | food selectivity towards unhealthy foods | food selectivity |
| **Situational (Individual)** | | |
| Hunger | food deprivation | food deprivation |
| Related health behaviours | related health behaviours*; individual food processing (cooking) | related health behaviours and occupational activity; individual food processing (cooking) |
| Situational and time constraints | need to shop outside locality (addition); situational or time constraints (incl. public transport and childcare) (addition) |  |
| **Social (Interpersonal)** | | |
| Family structure | family cohesion*; household size^✝^; single-parent household^✝^; equitable food distribution in family (addition) | family cohesion; household size; family composition |
| Household socioeconomic status | household food insecurity; food budget (addition); household resources | household food security |
| Social influence | social modelling of unhealthy eating; social relationships; stigma (addition) | peer modelling; social relationships |
| Social support | familial social support for diet | social support |
| Parental resources & risk factors | parental risk factors | parental risk factors (category level) |
| Parental attitudes & beliefs | parental healthy eating attitudes and belief | parental attitudes and beliefs (category level) |
| Parental behaviours | parental modelling and feeding | parental modelling |
| Parental feeding styles | authoritarian parenting styles (addition) |  |
| **Cultural (Interpersonal)** | | |
| Cultural cognitions | social model of consumption (addition); medical model of consumption (addition) |  |
| Cultural behaviours | food as membership to social group (addition) |  |
| **Product (Environment)** |  |  |
| Extrinsic product attributes | objective relative cost of healthy foods; acceptability of healthy food (addition) | price; brand |
| **Home and neighbourhood environment** | | |
| Home food availability & accessibility | availability of healthy food in household; accessibility of healthy food in household | home food availability and accessibility (category level) |
| Eating environment | characteristics of home (addition)* |  |
| Characteristics of living area | size of municipality*; negative perception of local area (addition); (impact of) accessible and affordable public transport or walkability (addition); residential or geographical segregation (addition); local market pressures (addition) | size of municipality |
| Environment food availability & accessibility | school canteen food environment*; availability of healthy food in local area; accessibility of healthy food in local area; perceived affordability of healthy food (addition); perception as captive customer in local area (addition) | school canteen food environment; neighbourhood healthy food availability |
| Food outlet density | fast food outlet density; supermarket and healthy food store density; | fast food outlet density; supermarket density and healthy food store density |
| Exposure to food promotion | exposure to promotions on food (addition) |  |
| **Food choice** | | |
| Percentage income on food | proportion of income on food budget (addition) |  |
| Willingness-to-pay | willingness-to-pay for healthy items; customer demand for healthy food (addition) | willingness-to-pay (category level) |
| Produce purchase | cost-determined purchases (addition); health-determined purchases; controlled purchases (addition) |  |
| **Eating behaviour** | | |
| Eating habits | strategic unhealthy eating habits (addition); disordered eating or overeating (addition) |  |

*overall non-significant association with other determinants, therefore not included in the systems map ^✝^treated as exposure rather than causal determinants
